# From hypercalcemia to the diagnosis of pseudohypoparathyroidism type 1b: a case report

**DOI:** 10.3389/fendo.2026.1778659

**Published:** 2026-03-10

**Authors:** Małgorzata Rumińska, Maria Krajewska, Agata Skórka, Ewelina Witkowska-Sędek

**Affiliations:** 1 Department of Paediatrics and Endocrinology, Medical University of Warsaw, Warsaw, Poland; 2 Department of Paediatrics, Medical University of Warsaw, Warsaw, Poland; 3 Department of Medical Genetics, The Children’s Memorial Health Institute, Warsaw, Poland

**Keywords:** case report, children, hyperphosphatemia, hypocalcemia, non-autoimmune hypothyroidism, parathyroid hormone, pseudohypoparathyroidism type 1B

## Abstract

Pseudohypoparathyroidism type 1b (PHP-1b) is a rare genetic disorder caused by mutations or epigenetic alterations of the maternal GNAS gene, resulting in isolated renal resistance to parathyroid hormone (PTH) and, in some cases, partial resistance to thyroid-stimulating hormone. Clinical manifestations typically result from hypocalcemia and resemble those seen in hypoparathyroidism, while laboratory tests show elevated serum PTH levels along with hypocalcemia and hyperphosphatemia.

We present a case of a girl with an unusual course of PHP: from non-autoimmune hypothyroidism coexisting with hypercalcemia at the age of 1 year to the diagnosis of PHP-1b at the age of 3.5 years, before the onset of symptomatic hypoparathyroidism.

In the absence of a clear cause for hypothyroidism, assessment of calcium and phosphate metabolism should be considered. Initial hypercalcemia does not exclude the diagnosis of PHP-1b. Long-term monitoring of calcium and phosphate metabolism parameters may be necessary for a final clinical diagnosis.

## Introduction

Pseudohypoparathyroidism type 1b (PHP-1b) belongs to the heterogeneous group of rare congenital disorders characterized by a lack of end-organ responsiveness to parathyroid hormone (PTH) and abnormalities in the PTH signaling pathway. It results from genetic defects or epigenetic modifications of the *GNAS* gene located in the chromosome 20q13.3 region, leading to loss of expression of the stimulatory G protein alpha subunit (Gsα) in renal proximal tubules (1). The Gsα protein activates adenylyl cyclase, which increases intracellular cyclic adenosine monophosphate (cAMP) levels, subsequently leading to the activation of protein kinase A (PKA). Dysregulation of the Gsα/cAMP/PKA pathway primarily affects the renal parathyroid hormone/parathyroid hormone-related peptide (PTH/PTHrp) signaling activated by the parathyroid hormone 1 receptor (PTHR1), which causes isolated renal PTH resistance with varying severity. It may also affect the thyroid-stimulating hormone (TSH), epinephrine, and calcitonin signaling (1–4). Expression of the *GNAS* gene is regulated by parental imprinting. In several tissues, including the proximal renal tubules, thyroid, gonads, and pituitary gland, expression of the *GNAS* gene is predominantly derived from the maternal allele, whereas the paternal allele is silenced. The inactivating mutation affecting the maternal allele of the *GNAS* gene (typical for PHP-1a) results in the full disease phenotype characterized by multihormonal resistance. Patients may exhibit hypothyroidism, hypogonadism, and growth hormone deficiency. In PHP-1b, genetic disorders concern the methylation regulating the expression of the maternal allele *GNAS*. These epigenetic alterations result in impaired *GNAS* expression predominantly in the renal proximal tubules, leading to renal resistance to PTH (5, 6).

Typical manifestations of PHP result primarily from hypocalcemia and resemble those seen in hypoparathyroidism, whereas laboratory tests reveal markedly elevated serum PTH levels, along with hypocalcemia and hyperphosphatemia. Patients may manifest neuromuscular irritability, including paresthesia, muscle cramps, and tetany, as well as seizures in more severe cases. Chronic hypocalcemia can lead to neuropsychiatric symptoms, cataracts, and ectopic calcifications.

In contrast to other forms of PHP, such as PHP-1a and PHP-1c, children with PHP-1b do not exhibit features of Albright hereditary osteodystrophy (AHO) or other endocrine abnormalities, although TSH resistance may be present (1, 2). Typical features of AHO include short stature, obesity, subcutaneous ossifications, brachydactyly with shortening of the third, fourth, and fifth metacarpals and/or metatarsals, shortening and broadening of the first phalanx of the thumb, round face, short neck, dental hypoplasia, skeletal deformities, and variable degrees of intellectual disability (2).

We present a case of a girl illustrating the unusual clinical course of PHP-1b, in whom the diagnosis of pseudohypoparathyroidism was preceded by a period of hypercalcemia.

## Case presentation

A girl aged 1 year and 4 months was referred to the Department of Paediatrics and Endocrinology due to subclinical hypothyroidism (TSH 8.296 µIU/ml, RRs 0.66 - 4.65; free thyroxine (fT4) 1.0 ng/dl, RRs 0.88 - 1.54) and iron deficiency anemia persisting despite four months of oral iron supplementation.

The girl was born at 39 weeks of gestation by cesarean section with an appropriate-for-gestational-age birth weight of 3465g. Apgar scores were 6/7/9 at 1/5/10 minutes after birth, respectively. The pregnancy was complicated by gestational diabetes and hypothyroidism. After birth, the newborn exhibited transient respiratory adaptation disorders requiring passive oxygen support. Physiological jaundice was observed. Due to the occurrence of tongue protrusion from the 6th month of life, a neurological consultation was conducted, but no abnormalities were found. The child was also examined by a cardiologist due to episodes of breath-holding with cyanosis during crying. A chest X-ray was normal, and echocardiography revealed a patent foramen ovale. The child also tended to have constipation. Due to selective mutism, the girl was under the care of an otolaryngologist, neuropsychologist, and speech therapist. The family history was unremarkable.

Upon admission to the Department of Paediatrics and Endocrinology, the girl was in good general condition but appeared socially withdrawn. The physical examination revealed no significant abnormalities; the thyroid gland was not palpable, and psychomotor development was normal, except for a delay in speech development. Anthropometric parameters were as follows: height at the 66th percentile, weight, and body mass index (BMI) above the 97th percentile according to World Health Organization (WHO) charts. Laboratory test results obtained upon admission (**Table 1**) showed elevated TSH, decreased fT4, normal free triiodothyronine (fT3) levels, absence of antithyroid antibodies, microcytosis, vitamin D deficiency, elevated calcium (Ca) and phosphate (P) levels, normal fasting glucose, elevated aminotransferase activity, and normal kidney function. Ultrasound of the thyroid gland and the abdomen revealed no abnormalities. The girl was discharged from the hospital with a recommendation to administer levothyroxine (LT-4) at a dose of 12.5 µg/day, vitamin D at a dose of 1000 IU/day, and oral iron supplementation at a dose of 5 mg/kg/day.

**Table 1:** Admission laboratory findings.

Parameters	Patient’s value	Reference range
TSH (µIU/ml)	9.81	0.66 - 4.65
Free T4 (ng/dl)	0.8	0.88 - 1.54
Free T3 (pg/ml)	3.53	2.77 - 4.71
Anti-Tg (IU/ml)	2.8	< 4.1
Anti-TPO (IU/ml)	0	< 5.6
RBC (cells x 10^6^/µl)	4.79	4.2 - 5.5
Hgb (g/dl)	11.2	11.0 - 14.0
MCV (fL)	71.4	72 - 88
TIBC (µg/dl)	471	184 - 377
Fe (µg/dl)	55	20 - 140
25(OH)D (ng/ml)	16.8	20 - 50
Calcium (mg/dl)	10.4	8.7 - 9.8
Phosphate (mg/dl)	6.7	3.9 - 6.5
Fasting glucose (mg/dl)	75	70 - 99
AST (U/l)	82	20 - 60
ALT (U/l)	78	5 - 45
Creatinine (mg/dl)	0.3	0.2 - 0.7

TSH, Thyroid-stimulating hormone; Free T4, free thyroxine; Free T3, free triiodothyronine; Anti-Tg, antibodies against thyroglobulin; Anti-TPO, antibodies against thyroid peroxidase; RBC, red blood cells; Hgb, hemoglobin; MCV, mean corpuscular volume; TIBC, Total Iron-Binding Capacity; Fe, iron; 25(OH)D, 25-hydroxyvitamin D; AST, aspartate aminotransferase; ALT, alanine aminotransferase.

**Figure 1:**
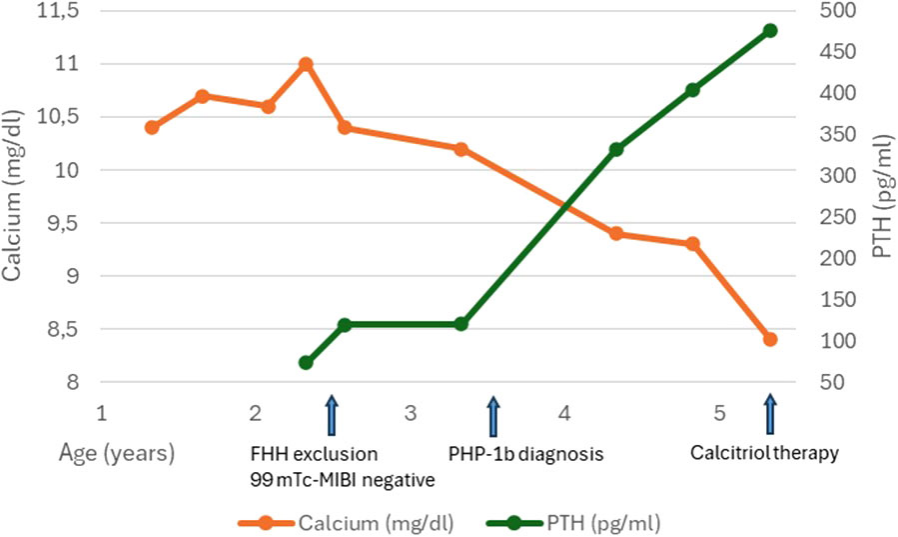
Changes in serum calcium and PTH levels.

After 4 months of LT-4 therapy, thyroid function parameters tended to normalize, and LT-4 substitution was continued with a gradually increasing dose up to 37.5 µg/day. Serum 25-hydroxyvitamin D [25(OH)D] concentration decreased due to irregular vitamin D supplementation. Surprisingly, the decrease in 25(OH)D levels coincided with a significant increase in serum Ca concentration and normalization of P levels (**Table 2**, **Figure 1**).

**Table 2:** Changes in calcium-phosphorus metabolism.

Parameters	Patient’s value									Reference range
	(Observation time/Patient’s age)									
	Baseline	4m	9 m	1y	1.25y	2y	3y	3.5y	4y	
	(1y4m)	(1y8m)	(2y1m)	(2y4m)	(2y7m)	(3y4m)	(4y4m)	(4y10m)	(5y4m)	
25OHD (ng/ml)	16.8	5.7	18	34.4	22.2	19.1	8.8	48.4	27	20 - 50
Calcium (mg/dl)	10.4^*^	10.7^*^	10.6^*^	11.0^*^	10.4^*^	10.2^**^	9.4^***^	9.3^***^	8.4^***^	^*^8.7 - 9.8
										^**^8.9 - 10.1
										***8.8 - 10.8
Phosphate (mg/dl)	6.7^*^	6.0^*^	6.0^*^	6.1^*^	6.0^*^	5.2^**^	6.1^***^	6.3^***^	6.0^***^	^*^3.9 - 6.5
										^**^4.0 - 5.4
										^***^3.2 - 5.5
ALP (U/l)	–	–	–	202.0^*^	–	215.0^**^	305.0^***^	306.0^***^	292.0^***^	^*^129 - 291
										^**^ 134 - 346
										^***^142 - 335
PTH (pg/ml)	–	–	–	73.5	119	120	332	405	477	12 - 95
Creatinine (mg/dl)	0.3	–	–	0.3	–	0.3	0.34	–	–	0.2 - 0.7
Albumin (g/dl)	–	–	–	4.6^*^	–	4.5^**^	4.7^***^	–	–	^*^3.4 - 4.2
										^**^3.5 - 5.2
										^***^3.8 - 5.4
FECa	–	–	–	< 0.0006	0.00042	0.0006	–	–	–	–
				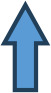		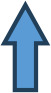			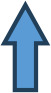		
				FHH exclusion 99mTc-MIBI negative		PHP-1b diagnosis			Calcitriol therapy		

25(OH)D, 25-hydroxyvitamin D; ALP, alkaline phosphatase; PTH, parathormone; FECa, fractional urinary calcium excretion.

*, **, *** - age-appropriate reference values.

In the following months (**Table 2**, **Figure 1**, results after 9 months and 1 year of follow-up), serum Ca levels gradually increased to 11.0 mg/dl, and serum 25(OH)D levels reached low-normal values (34.4 ng/ml). Detailed evaluation of Ca-P metabolism revealed high-normal PTH level (73.5 pg/ml, RRs: 12–95 pg/ml), normal alkaline phosphatase (ALP) activity (202 U/l, RRs: 129–291 U/l), and low fractional urinary Ca excretion (FECa) (<0.0006). Genetic testing was conducted to identify loss-of-function mutations in the calcium-sensing receptor (CaSR) gene, which is responsible for familial hypocalciuric hypercalcemia (FHH). The test ruled out FHH when the child was 2 years and 5 months old.

Further evaluation (**Table 2**, **Figure 1**, results after 1.25 years, 2 years, 3 years, and 3.5 years of observation) revealed increasing PTH levels with a tendency toward spontaneous normalization of serum Ca levels, normal but increasing P levels, and low FECa. Liver and renal function parameters, as well as albumin levels, remained within the normal range. Differential diagnosis included multiple imaging tests. Abdominal and thyroid ultrasound were repeated but revealed no significant abnormalities. Parathyroid scintigraphy performed at the age of 2 years and 7 months, using a dual-phase protocol with 99mTc-MIBI at a dose of 8 mCi, with SPECT performed 15 minutes after tracer administration and SPECT/CT (DLP 22) performed 3 hours later, did not reveal any features suggestive of parathyroid adenoma. No abnormal foci of tracer uptake were observed in the cervical or mediastinal area. Total body densitometry showed bone mineral density within the narrow age-specific reference range (Total Body z-score: -0.6).

At the age of 3.5 years, methylation-specific multiplex ligation-dependent probe amplification (MS-MLPA) analysis of the *GNAS* gene was performed using the SALSA MS-MLPA probemix ME031 GNAS (MRC Holland). Epigenetic testing revealed hypermethylation of the NESP differentially methylated region (NESP-DMR) and hypomethylation of the A/B-DMR, XL-DMR, and AS1-DMR, supporting the diagnosis of PHP-1b (OMIM#603233). Any chromosomal imbalances were excluded.

After 4 years of follow-up, Ca-P metabolism parameters became typical of PHP: Ca levels decreased, and PTH levels reached a peak of 477.0 pg/ml. At the age of 5 years and 4 months, the child started calcitriol therapy at an initial dose of 0.25 µg daily, along with oral calcium supplementation of 350 mg. Vitamin D supplementation was continued at a dose of 2000 IU daily. A low-phosphate diet was also recommended.

Currently, the girl has been receiving calcitriol for 2 years. The dose of the medication (calcitriol, calcium, and vitamin D) remains unchanged, and she also receives LT-4 at a dose of 1.87 µg/kg/day. Her physical and psychomotor development is normal. Her height, weight, and BMI are in the 75th percentile. Current values for Ca-P metabolism are presented in **Table 3**.

**Table 3:** Actual on-treatment laboratory findings.

Parameters	Patient’s value	Reference range
TSH (µIU/ml)	2.933	0.58 - 3.59
Free T4 (ng/dl)	1.02	0.84 - 1.47
Free T3 (pg/ml)	4.11	2.85 - 4.44
25(OH)D (ng/ml)	30.5	20 - 50
1,25(OH)_2_D (pg/ml)	69.1	45 - 102.5
Calcium (mg/dl)	9.7	8.8 - 10.8
Ionized Calcium (mmol/l)	1.11	1.16 - 1.31
Phosphate (mg/dl)	5.9	3.1 - 5.5
Magnesium (mg/dl)	2.2	1.7 - 2.1
ALP (U/l)	325	142 - 335
PTH (pg/ml)	348.2	13.3 - 72.8
Creatinine (mg/dl)	0.43	0.4 - 0.6

TSH, Thyroid-stimulating hormone; Free T4, free thyroxine; Free T3, free triiodothyronine; 25(OH)D, 25-hydroxyvitamin D; 1,25(OH)_2_D, 1,25-dihydroxyvitamin D; ALP, alkaline phosphatase; PTH, parathormone.

## Discussion

We present the case of a girl with an unusual course of PHP-1b. Serum Ca and 25(OH)D levels were routinely assessed as part of the diagnostic workup for thyroid dysfunction. Blood tests revealed hypothyroidism without signs of autoimmunity, along with elevated serum Ca levels. After nearly two years of persistent, stable hypercalcemia, changes in Ca-P metabolism prompted further diagnostic workup, ultimately leading to the diagnosis of PHP-1b.

Initially, hypersensitivity to vitamin D, resulting in hypercalcemia with elevated P levels, was considered a possibility. However, the low baseline 25(OH)D concentration and the lack of a decrease in serum Ca concentration after discontinuing vitamin D supplementation effectively ruled out this possibility. Elevated PTH levels further contradicted this hypothesis.

Physical examination revealed no dysmorphic features, ruling out congenital causes of hypercalcemia, such as Williams Syndrome.

The absence of clinical symptoms of mild, non-progressive hypercalcemia, combined with mildly elevated PTH levels, **Table 2** 5(OH)D levels, and inappropriately low urinary Ca excretion relative to hypercalcemia, suggested FHH. Distinguishing FHH from primary hyperparathyroidism (PHPT) includes measurement of the FECa. In most patients with FHH, FECa is less than 0.01, although in more than 20% of cases, FECa may exceed 0.01. Approximately 65% of FHH cases are caused by inactivating mutations in the *CaSR* gene (FHH1), a mutation that was not detected in our patient. Mutations in other genes involved in calcium signaling, including *GNA11* and *AP2S1*, which are responsible for FHH2 and FHH3, respectively, were not assessed at that time (7).

Although a parathyroid abnormality was considered unlikely, the progressive increases in PTH levels, despite mild and stable hypercalcemia and low FECa levels, prompted further evaluation for a possible parathyroid tumor. Parathyroid adenomas occur sporadically in children, and primary hyperparathyroidism is most often caused by a single benign adenoma, accounting for approximately 65-70% of cases (8). Our patient underwent dual-phase Tc-MIBI scintigraphy with single-photon emission computed tomography/computed tomography (SPECT/CT), which revealed no abnormalities.

In the third year of observation, a gradual decrease in serum Ca concentration was noted, accompanied by a gradual increase in P concentration and a marked increase in PTH levels, suggesting PHP. The absence of dysmorphic features and skeletal symptoms indicated PHP-1b.

In most cases, the diagnosis of PHP-1b is prompted by symptoms of hypocalcemia (9–14). PTH resistance develops gradually over time. In children, symptoms usually appear after the age of 5, during periods of rapid growth, which is likely related to an increased need for calcium and vitamin D (3, 13, 15). The literature review we conducted confirmed these observations - most of the described patients with PHP-1b were between 10 and 14 years old (9–11). To the best of our knowledge, the youngest reported patient was 2 years and 3 months old (14). Only isolated reports are available of patients whose disease began in adulthood (10–12). In individual patients, the diagnosis is made incidentally due to an isolated increase in PTH levels with normocalcemia. Initially, this usually raises suspicion of normocalcemic primary or secondary hyperparathyroidism before the correct diagnosis of PHP-1b is made (6).

Our patient exhibited a rare course of PHP-1b, initiated by hypercalcemia. Long-term observation of Ca-P metabolism parameters allowed for establishing the correct diagnosis.

In the literature, we did not find any case reports of children with PHP-1b who presented with hypercalcemia. Shalitin et al. (9) described the diversity in the clinical presentation and course of PHP-1 in 8 children who underwent long-term follow-up. Hypercalcemia was observed in one of the described children; however, due to the lack of genetic testing, it was not confirmed that this was PHP-1b. The authors also reported that relative hypocalciuria was a rule both at the time of diagnosis and during PHP-1 therapy. They recommend regular monitoring of PTH levels in cases of Ca-P metabolism disorders of unclear etiology (9).

In some individuals with PHP-1b, PTH resistance may be accompanied by partial TSH resistance, with slightly elevated TSH levels and normal or low serum thyroid hormone levels. Moreover, in many cases, similar to the girl reported here, TSH resistance may precede the development of PTH resistance by several years (12). The subsequent diagnosis of PHP-1b definitively clarifies the cause of hypothyroidism. Other symptoms, such as mild brachydactyly or Madelung-like defect, excessive growth, and weight gain in early infancy, have also been reported in individuals with PHP-1b. Intellectual development is usually normal in those patients (1, 16).

The wide spectrum of clinical manifestations observed between the two forms of PHP type 1 (1a and 1b) may be explained, at least in part, by haploinsufficiency of Gαs in specific tissues (15, 17). Typically the renal PTH resistance is located in the proximal renal tubule, where 1-α-hydroxylase converts 25(OH)D to 1,25(OH)_2_D. Low 1,25(OH)_2_D levels lead to hypocalcemia, as 1,25(OH)_2_D is necessary for intestinal calcium absorption (18). However, it remains unclear why some patients with PHP-1b exhibit transient hypercalcemia, whereas others remain normocalcemic. One possible explanation is that epigenetic defects of the GNAS locus, leading to variable Gsα expression in the kidneys, account for the heterogeneous resistance to PTH, even among renal cells. This could reflect partial or delayed renal resistance to PTH in early life. Residual Gsα function in renal cells may allow for the transient maintenance of normocalcemia or hypercalcemia. Over time, as PTH resistance becomes more pronounced, the characteristic biochemical features of PHP-1b gradually emerge. Paradosu et al. (12) suggested that normocalcemia in patients with PHP-1b may result from preserved PTH action on other target tissues, such as bone and the distal renal tubules, which could help maintain normal serum calcium levels.

The girl we described belongs to approximately 80% of patients with the sporadic form of PHP-1b, as evidenced by the characteristic methylation pattern (19). The *GNAS* gene is an imprinted locus with four epigenetically regulated promoters, and one paternally differentially methylated region (GNAS-NESP: TSS-DMR) and three maternally methylated DMRs (GNAS-AS1: TSS-DMR, GNAS-XL: Ex1-DMR, and GNAS A/B: TSS-DMR). Loss of methylation at the GNAS A/B: TSS-DMR is a key epigenetic hallmark of PHP-1b. In sporadic cases, epigenetic alterations usually involve multiple GNAS DMRs. In a small portion of patients (approximately 8-10%), methylation abnormalities result from paternal uniparental disomy involving part or all of chromosome 20q. In the remaining sporadic cases, the underlying genetic or epigenetic mechanism remains unknown (17, 19, 20).

Considering the heterogeneity of clinical manifestations of PHP, genetic testing should be more widely used in patients with disturbances of Ca-P metabolism. The diagnosis of PHP-1b can be particularly challenging, especially in the absence of typical biochemical abnormalities such as hypocalcemia or hyperphosphatemia. Early molecular diagnosis allows for the timely initiation of treatment, potentially preventing the development of symptomatic hypocalcemia.

## Conclusions

The initial clinical manifestation of PHP-1b may be atypical. Persistent, unexplained hypercalcemia does not exclude subsequent diagnosis of PHP-1b and requires long-term monitoring with regular assessment of PTH. In cases of hypothyroidism of unclear etiology, an evaluation of Ca-P metabolism should be considered.

## Data Availability

The raw data supporting the conclusions of this article will be made available by the authors, without undue reservation.
